# Emotional distress was associated with persistent shoulder pain after physiotherapy: a prospective cohort study

**DOI:** 10.1186/s12891-018-2142-3

**Published:** 2018-08-22

**Authors:** Kaja Smedbråten, Britt Elin Øiestad, Yngve Røe

**Affiliations:** Department of Physiotherapy, Faculty of Health Sciences, OsloMet – Oslo Metropolitan University, Pb 4, St.Olavs plass, 0130 Oslo, Norway

**Keywords:** Shoulder pain, Emotional distress, Physiotherapy

## Abstract

**Background:**

There is a paucity of research on the association between psychological factors and persistent shoulder pain. The aim of this study was to investigate whether emotional distress was associated with pain intensity and self-reported disability after physiotherapy treatment in patients with shoulder pain.

**Methods:**

Data from 145 patients treated at physiotherapy outpatient clinics aged ≥18 years with self-reported pain in the shoulder or arm, and movement activity problems related to the upper-extremity, were included. Outcome measures were pain intensity measured by Numeric Pain Rating Scale and disability measured by Patient Specific Functional Scale. Demographic and clinical characteristics, including emotional distress measured by Hopkins Symptom Checklist – 25, were obtained at study onset. Association between characteristics at study onset and pain and disability after physiotherapy treatment were analysed using multiple linear regression and a backward manual elimination method. The final models were adjusted for age and sex.

**Results:**

Higher emotional distress at study onset (B 1.06, 95% CI 0.44 to 1.68) was associated with higher pain intensity after the physiotherapy treatment (*P* = 0.001). Emotional distress was not associated with self-reported disability after the physiotherapy treatment.

**Conclusion:**

This study found that emotional distress at study onset was associated with shoulder pain intensity after physiotherapy treatment, but not with disability. The findings indicate that emotional distress should be included in the initial physiotherapy examination of shoulder pain.

**Electronic supplementary material:**

The online version of this article (10.1186/s12891-018-2142-3) contains supplementary material, which is available to authorized users.

## Background

Shoulder pain is a common disorder in the general population, with a point prevalence ranging from 6.9 to 26%, and a lifetime prevalence from 6.7 to 66.7% [1]. In many patients, the shoulder pain is long lasting, and 41% of the patients report persistent symptoms one year after they initially sought help for their problem [2]. Exercise therapy is a common treatment modality for shoulder pain, and there is evidence to support that physiotherapist-prescribed exercise decreases pain and improves function at short-term follow-up [3, 4]. However, the evidence of its long-term effectiveness has been questioned [5].

A systematic review of prognostic factors in patients with acute and subacute non-traumatic shoulder pain found strong evidence that high scores on the Shoulder Pain and Disability Index (SPADI), more shoulder pain, and a longer duration of complaints were associated with persistent shoulder pain [6]. Moderate evidence was found for male gender, age > 55 years, poor general health, a gradual onset of complaints, longer duration of sick leave, the perception of high job demand, low perceived social support, and the number of visits to a general practitioner [6]. The authors of the review suggested that the lack of identified psychosocial prognostic factors could be due to little use of questionnaires containing these functions in shoulder pain populations [6].

A recent cohort study, which included a range of biopsychosocial factors, found that psychological factors were consistently associated with the outcome of physiotherapy for patients with shoulder pain [7]. The psychological factors that were associated with a better outcome at six weeks and six months were higher pain self-efficacy and patient expectations of ‘complete recovery’ in comparison to ‘slight improvement’ as a result of physiotherapy treatment [7]. The association between emotional factors and the outcome of physiotherapy in patients with shoulder pain has been scarcely investigated in epidemiological research. The aim of this study was to investigate whether emotional distress was associated with pain intensity and self-reported disability after physiotherapy treatment in patients with shoulder pain.

## Methods

This study was a prospective cohort study of consecutive patients treated at two student clinics located at the Department of Physiotherapy at OsloMet – Oslo Metropolitan University in Norway between September 2013 and September 2016. Patients receiving physiotherapy at the two clinics answered questionnaires for the FysioPol database. The FysioPol database contains pre- and post-treatment information about the patients treated at the student clinics, and was established in order to measure the quality of treatment and facilitate research at the department [8]. The database includes information on socio-demographic status and characteristics of the patients’ complaints such as pain duration, pain intensity, disability, medication and emotional distress. The data is collected through electronic questionnaires.

The patients were treated by physiotherapy students in their second or third study year, under supervision of a teacher. The treatment period was intended to be up to nine weeks. The treatment consisted of individualised exercise therapy. In addition, some of the patients reported that they had received information, advice and manual techniques such as massage and stretching.

Patients aged 18 years or older with self-reported pain in the shoulder or arm were included. We excluded patients who did not report any movement activity problems related to the upper-extremity in the Patient Specific Functional Scale (PSFS) [9]. Patients who were unable to read and understand Norwegian were excluded.

The study protocol was considered by the Regional Committee for Medical and Health Research Ethics in Norway (REC), which concluded that the study did not require ethical approval. The study was approved by the Norwegian Centre for Research Data (NSD). All patients had signed a written, informed consent form.

### Outcome measures

The primary outcome in this study was the Numeric Pain Rating Scale (NPRS) [10]. The NPRS is an 11 point scale where 0 indicates no pain, and 10 indicates the worst imagined pain. In this study, the scale was used as a measure of pain intensity during the last week. The PSFS was used as a secondary outcome [9]. In this questionnaire, the patients wrote down up to three activities they found impossible or had difficulty doing because of their problem. The difficulty associated with each activity was rated on a scale from 0 (impossible to perform the activity) to 10 (no difficulty, or at the same level as before the pain occurred). The average rating of the activities was used in the analyses as a measure of self-reported disability [11, 12]. Only scores from activities that had been rated both before and after treatment were included in the calculation of the average score.

### Potential prognostic factors

The set of variables considered as potential prognostic factors was obtained by the FysioPol-questionnaire package at study onset, including clinical characteristics identified with prognostic value in previous research, and demographic factors. The Hopkins Symptom Checklist-25 (HSCL-25) [13] was used as a measure of emotional distress. The questionnaire aims to assess symtomps of anxiety, depression and somatization. HSCL-25 is a shorter version of the Symptom Checklist 90 (SCL-90) and consists of 25 items that are rated from 1 (not at all) to 4 (very much). The total score was obtained by averaging the scores, and ranged between 1 and 4. A maximum of five missing items were accepted. A higher total score indicates a higher level of emotional distress. The Norwegian version of the HSCL-25 has been used in several studies of musculoskeletal pain [14–17]. Evidence of psychometric properties of HSCL-25 in the population of patients with shoulder pain is to our knowledge lacking. Other clinical characteristics considered as potential prognostic factors included pre-treatment pain intensity measured by NPRS; pre-treatment disability measured by PSFS; duration of pain divided into 0–3 months, 4–12 months and more than 12 months; use of pain relieving drugs divided into less than every week and every week or more; concomitant neck pain; and number of pain sites divided into two pain sites or less and three pain sites or more. The demographic factors included age, sex, body mass index (BMI), level of education, work status, relationship status and smoking status. Level of education was divided into lower level (≤ 13 years) and college / university (> 13 years). Work status was divided into working and not working. Students were included in the working group, which consisted of both full time and part time working patients, while retirees were included in the not working group, which also included unemployed patients and patients on full time sick leave and disability pension.

### Statistical analyses

Descriptive data are presented as number of patients and percentages, means and standard deviations or medians and interquartile range. Paired t-tests were used to identify changes from pre- to post- treatment measures of pain intensity and self-reported disability. Characteristics of the individuals who were lost to follow-up were compared to pre-treatment characteristics of the study sample. The groups were compared on pain intensity, disability, emotional distress and age using independent t-tests, and sex using a Chi-square test.

Simple linear regression analyses were performed to examine the relationship between each of the potential prognostic factors and the outcome (the NPRS and the PSFS). The variables with a statistically significant relationship with the outcome at the 20% level (*P* < 0.20) [18] were considered for the final multiple regression models. A backward manual elimination method was used to remove those variables with the highest *P*-value, one by one. The elimination was repeated until the remaining variables in the models were all statistically significant at the 5% level (*P* < 0.05). To prevent elimination of a variable at one step in the analysis process being crucial, the variables removed on backward elimination were all re-entered in the models one by one, and remained in the models if they were statistically significant at the 5% level. The multiple regression models were adjusted for age and sex.

Assumptions for the regression models were assessed. Correlation analyses were performed for all the independent variables and the correlation had to be less than 0.7 between the variables to be entered in the models [18]. An extreme value in the variable of BMI (> 39) was interpreted as an univariate outlier, due to a standardized score in excess of 3.29, disconnected from the other standardized scores [18]. Since a BMI-value of > 39 may involve other health problems than the ones investigated in this study, and the case therefore may not be a part of the population we intended to investigate, the case was excluded.

SPSS version 24 was used for all of the statistical analyses.

## Results

**Fig. 1:**
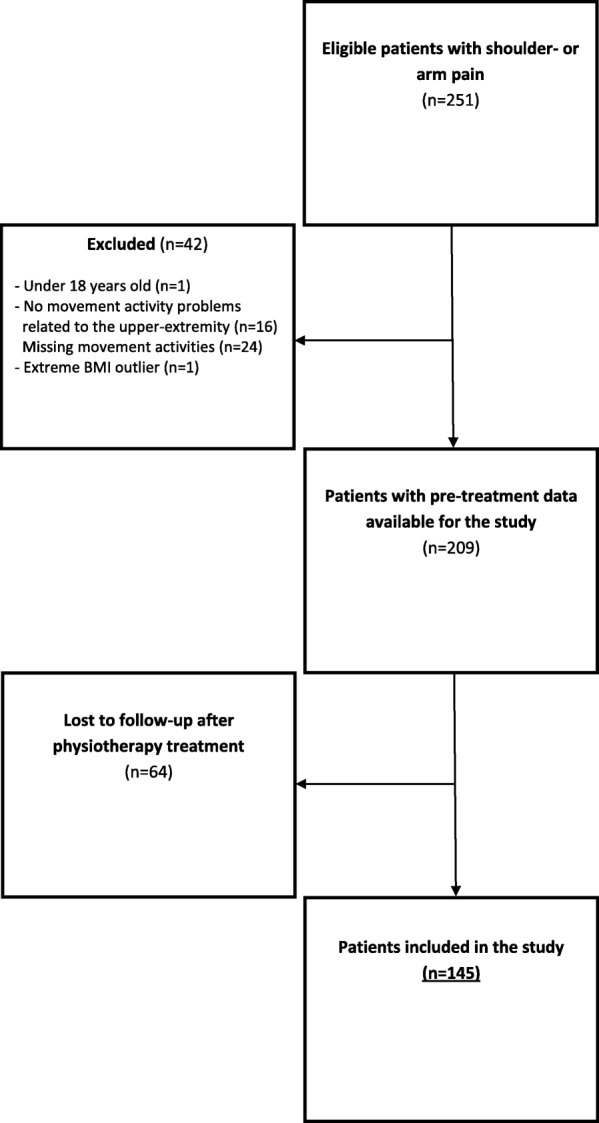
Flow chart of the study

Altogether, 251 patients reported shoulder- or arm pain during the inclusion period and were eligible for participation (Fig. 1). Of these, 209 patients met the inclusion criteria, but 30.6% did not answer the post- treatment questionnaires. Thus, 145 patients were included in the study. The patients lost to follow-up did not differ in age, sex, pain intensity, disability or level of emotional distress at study onset compared to the study sample (*P* > 0.05) (see Additional file 1 Table S1). The demographic and clinical characteristics of the patients at study onset are shown in Table 1. The study group had an average level of emotional distress of 1.6 (SD 0.5). The average pre-treatment pain intensity was 4.9 (SD 2.3), and the average self-reported disability was 4.5 (SD 2.0).

**Table 1:** Characteristics of the study sample at study onset (*n* = 145)

Variables	Frequency (%)	Mean (SD)
Age		44.0 (15.4)
Sex, female (missing: 2)	104 (71.7)	
BMI (missing: 25)		24.7 (3.7)
Education		
≤ 13 years	45 (31.0)	
College /university	98 (67.6)	
(Missing: 2)		
Work status		
Working or being in education	119 (82.1)	
Not working	26 (17.9)	
Relationship status		
In a relationship	75 (51.7)	
Not in a relationship	68 (46.9)	
(Missing: 2)		
Smoking (missing: 2)	12 (8.3)	
Emotional distress (HSCL-25)^a^ (1–4)		1.6 (0.5)
Pain intensity (NPRS)^b^ (0–10) (Missing: 18)		4.9 (2.3)
Disability (PSFS)^c^ (0–10)		4.5 (2.0)
Duration of pain		
0–3 months	28 (19.3)	
4–12 months	39 (26.9)	
> 12 months	78 (53.8)	
Use of pain relieving drugs		
Every week or more	31 (21.4)	
Less than every week	113 (77.9)	
(Missing: 1)		
Concomitant neck pain	60 (41.4)	
Number of pain sites		
> 2 sites of pain	24 (16.6)	
≤ 2 sites of pain	121 (83.4)	

^a^ HSCL-25 = Hopkins Symptom Checklist – 25, the average of 25 questions rated between 1: not at all, 4: very much

^b^ NPRS = Numeric Pain Rating Scale, 0: no pain, 10: worst imagined pain

^c^ PSFS = Patient Specific Functional Scale, the average of up to three activities rated between 0: impossible to perform the activity, 10: no difficulty, or at the same level as before the pain occurred

The median length of the treatment period was 5 weeks (IQR 3 to 6) (*n*=112). The patients had a statistically significant improvement in pain intensity from pre- to post-treatment (*P* < 0.001) of 2.0 (SD 1.9) points. The patients had also a statistically significant improvement in self-reported disability (P < 0.001) of 1.7 (SD 2.7) points. The average post-treatment pain intensity was 2.9 (SD 2.1) (*n*=140) and the post-treatment self-reported disability was 6.2 (SD 2.6) (*n*=133).

The results from the simple linear regression analyses between potential prognostic factors and pain intensity after treatment are presented in Table 2. A number of factors showed a statistically significant association at the 20% level with pain intensity after treatment. These were: emotional distress, pre-treatment pain intensity, pre-treatment disability, duration of pain for more than 12 months in comparison to 0 to 3 months, use of pain relieving drugs, concomitant neck pain, number of painful sites, sex, BMI and work status. In the final multiple model, higher emotional distress, higher pre-treatment pain intensity and duration of pain for 4 to 12 months in comparison to 0 to 3 months were associated with higher pain intensity after treatment (Table 2).

**Table 2:** Linear regression of pain intensity after treatment (NPRS) and potential prognostic factors

	Simple regression		Final multiple regression model ^b^ *N=122*	
	B (95% CI)	*P*-value	B (95% CI)	*P*-value
Emotional distress (HSCL-25)	2.05 (1.49 to 2.60)	*P* < 0.001	1.06 (0.44 to 1.68)	*P* = 0.001
Pre-treatment pain intensity (NPRS)	0.58 (0.45 to 0.71)	*P* < 0.001	0.46 (0.32 to 0.61)	*P* < 0.001
Pre-treatment disability (PSFS)	−0.22 (− 0.39 to − 0.05)	*P* = 0.012		
Duration of pain				
0–3 months ª				
4–12 months	0.34 (−0.72 to 1.39)	*P* = 0.528	0.93 (0.07 to 1.78)	*P* = 0.033
> 12 months	0.75 (− 0.18 to 1.69)	*P* = 0.112	0.75 (− 0.01 to 1.50)	*P* = 0.053 ^c^
Use of pain relieving drugs (0,1)	1.62 (0.78 to 2.46)	*P* < 0.001		
Concomitant neck pain (0,1)	1.34 (0.65 to 2.03)	*P* < 0.001		
Number of painful sites (0,1)	1.09 (0.16 to 2.02)	*P* = 0.022		
Age	−0.01 (− 0.03 to 0.02)	*P* = 0.508		
Sex (0,1)	0.88 (0.12 to 1.64)	*P* = 0.024		
BMI	0.09 (−0.02 to 0.20)	*P* = 0.100		
Education (0,1)	−0.45 (− 1.22 to 0.32)	*P* = 0.250		
Work status (0,1)	−0.99 (− 1.90 to − 0.09)	*P* = 0.032		
Relationship status (0,1)	0.00 (− 0.70 to 0.70)	*P* = 0.997		
Smoking (0,1)	0.77 (−0.50 to 2.05)	*P* = 0.233		

Duration of treatment was not associated with pain intensity after treatment (*P* = 0.951)

^a^ Reference category

^b^ Adjusted for age and sex

^c^ Before the model was adjusted for age and sex, duration of pain > 12 months was associated with pain intensity after treatment (*P* < 0.05)

NPRS = Numeric Pain Rating Scale. HSCL-25 = Hopkins Symptom Checklist – 25. PSFS = Patient Specific Functional Scale

Use of pain relieving drugs (0: < once a week, 1: ≥ once a week). Concomitant neck pain (0: no, 1: yes). Number of painful sites (0: ≤ 2 painful sites, 1: > 2 painful sites). Sex (0: male, 1: female). Education (0: ≤ 13 years, 1: College / University). Work status (0: not working, 1: working full time, part time or being in education). Relationship status (0: not in a relationship, 1: in a relationship). Smoking (0: no, 1: yes)

The results of the simple linear regression analyses between potential prognostic factors and disability after treatment (PSFS) are presented in Table 3. A number of factors showed a statistically significant association at the 20% level with disability after treatment. These were: emotional distress, pre-treatment pain intensity, pre-treatment disability, duration of pain for more than 12 months in comparison to 0 to 3 months, use of pain relieving drugs, concomitant neck pain, number of painful sites, age, education, work status and smoking status. In the final multiple model, higher pre-treatment disability, duration of pain for more than 12 months in comparison to 0 to 3 months, concomitant neck pain and a lower level of education (≤ 13 years) were associated with higher self-reported disability after treatment (Table 3).

**Table 3:** Linear regression of disability after treatment (PSFS) and potential prognostic factors

	Simple regression		Final multiple regression model ^b^ *N = 130*	
	B (95% CI)	*P*-value	B (95% CI)	*P*-value
Emotional distress (HSCL-25)	− 1.62 (− 2.43 to − 0.81)	*P* < 0.001		
Pre-treatment pain intensity (NPRS)	−0.16 (− 0.36 to 0.04)	*P* = 0.123		
Pre-treatment disability (PSFS)	0.45 (0.24 to 0.66)	*P* < 0.001	0.32 (0.10 to 0.53)	*P* = 0.004
Duration of pain				
0–3 months ª				
4–12 months	−0.12 (−1.43 to 1.19)	*P* = 0.859	−0.60 (− 1.81 to 0.61)	*P* = 0.327
> 12 months	−1.23 (− 2.39 to − 0.06)	*P* = 0.039	−1.17 (− 2.24 to − 0.11)	*P* = 0.031
Use of pain relieving drugs (0,1)	−1.68 (− 2.72 to − 0.65)	*P* = 0.002		
Concomitant neck pain (0,1)	− 1.47 (−2.36 to − 0.59)	*P* = 0.001	−1.14 (− 2.01 to − 0.28)	*P* = 0.010
Number of painful sites (0,1)	−0.97 (− 2.15 to 0.21)	*P* = 0.106		
Age	−0.02 (− 0.05 to 0.01)	*P* = 0.191		
Sex (0,1)	−0.54 (− 1.53 to 0.45)	*P* = 0.283		
BMI	−0.08 (− 0.21 to 0.05)	*P* = 0.235		
Education (0,1)	1.20 (0.25 to 2.15)	*P* = 0.014	0.94 (0.03 to 1.84)	*P* = 0.042
Work status (0,1)	2.04 (0.90 to 3.18)	*P* = 0.001	1.13 (−0.15 to 2.41)	*P* = 0.083 ^c^
Relationship status (0,1)	0.04 (−0.87 to 0.95)	*P* = 0.928		
Smoking (0,1)	−1.64 (−3.19 to −0.08)	*P* = 0.039		

Duration of treatment was not associated with disability after treatment (*P* = 0.407)

^a^ Reference category

^b^ Adjusted for age and sex

^c^ Before the model was adjusted for age and sex, work status was associated with disability after treatment (*P* < 0.05)

PSFS = Patient Specific Functional Scale. HSCL-25 = Hopkins Symptom Checklist – 25. NPRS = Numeric Pain Rating Scale

Use of pain relieving drugs (0: < once a week, 1: ≥ once a week). Concomitant neck pain (0: no, 1: yes). Number of painful sites (0: ≤ 2 painful sites, 1: > 2 painful sites). Sex (0: male, 1: female). Education (0: ≤ 13 years, 1: College / University). Work status (0: not working, 1: working full time, part time or being in education). Relationship status (0: not in a relationship, 1: in a relationship). Smoking (0: no, 1: yes)

## Discussion

This study showed that higher emotional distress at study onset, in combination with higher pre-treatment pain intensity and duration of pain for 4 to 12 months in comparison to 0 to 3 months, was associated with a poor outcome in terms of pain intensity after physiotherapy in patients with shoulder pain. Emotional distress was not associated with self-reported disability.

### Pain outcome

In a recent systematic review of prognostic factors for shoulder pain, strong evidence was found that high scores on the SPADI questionnaire, more shoulder pain, and a longer duration of complaints, were associated with persistent shoulder pain [6]. In contrast to the results of the present study, the systematic review did not find any evidence that psychological factors were associated with shoulder pain. However, the authors of the systematic review claimed that psychosocial factors might have been underestimated due to limited use of questionnaires containing these functions in shoulder pain populations [6].

Emotional distress was investigated in two previous studies on shoulder pain, which found no association with outcome [16, 17]. One of the studies comprised patients with diagnosed subacromial pain in secondary care [16], the other study included patients with diagnosed rotator tendinosis in primary care [17]. The inconsistencies between these studies and the present findings might be explained by differences in the study populations; the present study was a cohort study comprising patients with self-reported shoulder or arm pain.

Furthermore, the present findings are not consistent with those in a previous study on patients presenting new episodes of shoulder- or low back pain to their general practitioner [19]. Interestingly, the cohort study found that for the shoulder pain patients, no psychological factors were associated with persistent symptoms or disability after three months, with the exception of catastrophizing, which in patients with a long duration of pain at study onset (≥ 3 months) was associated with persistent symptoms [19]. However, it is worth noting that the patients in this study had no distress at baseline, measured by a subscale of the Four-Dimensional Symptom Questionnaire [20], while the patients in our study had an average level of 1.6 (SD 0.5) on the HSCL-25. This might explain the difference in findings. Another possible reason for different results might be that factors associated with persistent pain after an exercise therapy intervention differ from factors associated with pain after other types of treatment.

Nevertheless, a recent cohort study from the UK reported that other psychological factors than distress, such as patient expectations of recovery and pain self-efficacy, were associated with the level of pain and disability after physiotherapy in patients with shoulder pain [7]. Although the study did not identify any association between anxiety and depression and outcome, the authors suggested that this could be due to a low number of included patients with extreme anxiety and depression [7]. Based on the findings of the study, the authors concluded that when assessing people with musculoskeletal shoulder pain and considering referral to physiotherapy services, psychosocial and medical information should be considered [7].

### Disability outcome

Our data showed no association between emotional distress at study onset and self-reported disability after treatment. The factors associated with higher post-treatment disability were higher pre-treatment disability, duration of pain for more than 12 months in comparison to 0 to 3 months, concomitant neck pain and a lower level of education. The results indicate that patients with a history of chronic shoulder pain and disability may have a poor outcome in terms of disability regardless of emotional distress.

Our findings that emotional distress was associated with pain, but not with disability, are difficult to explain. One explanation might be that mental functions are more directly associated with the experience of pain, than with disability. However, a cross-sectional study on people with chronic shoulder pain found that psychological distress was correlated with disability, but not with pain [21], which indicate that the relationship between distress, disability and pain may be complex.

### Limitations

This study has some limitations that should be considered. Firstly, since the inclusion of patients was based on self-reported shoulder- or arm pain, we were not able to discriminate between localised shoulder pain and pain related to the shoulder, arm and hand. Secondly, a number of patients who met the inclusion criteria were excluded from the analyses due to loss to follow-up or missing values in some of the variables. However, the patients lost to follow-up did not differ from the study sample on characteristics such as sex, age, pain intensity, disability or emotional distress at study onset. A third concern involves the duration of treatment. There was a variety in duration of treatment, with a median of 5 weeks of physiotherapy (IQR 3 to 6). A period of 12 weeks of physiotherapy is often suggested for patients with shoulder pain [22]. Future research could establish whether the results after a longer follow-up differ from the results in this study.

### Implications for practice and research

The results of this study showed that emotional distress at study onset was associated with the intensity of shoulder pain after physiotherapy. Psychological factors in general are little emphasised in the examination of shoulder pain, and it is rather the structural and biomechanical aspects of the condition that are usually considered in clinical decision-making. The findings of this study support, however, that emotional distress should be considered in the initial physiotherapy examination of shoulder pain.

Emotional distress may be a cause or a consequence of shoulder pain, and whether the treatment should be directed towards reducing distress is not possible to tell based on the present findings. Nevertheless, findings of a meta-analysis on neck and back pain indicate that psychological distress mediates the relationship between pain and disability [23]. Future research is needed to investigate the relationship between emotional distress, pain and disability. The research should identify whether targeting emotional distress in shoulder pain rehabilitation is likely to improve the outcome for shoulder patients with a high degree of emotional distress, to identify whether and how to best individualise the treatment for these patients.

## Conclusion

This study found that higher emotional distress, in combination with higher pain intensity and duration of pain for 4 to 12 months in comparison to 0 to 3 months, was associated with a poor outcome in terms of shoulder pain intensity after physiotherapy, but not with disability. The present findings indicate that emotional distress should be included in the initial physiotherapy examination of shoulder pain.

## Additional file

Additional file 1: **Table S1.** Characteristics of the group lost to follow-up compared to the study sample. (DOCX 15 kb)

## Abbreviations

HSCL-25: Hopkins Symptom Checklist – 25
NPRS: Numeric Pain Rating Scale
PSFS: Patient Specific Functional Scale
SPADI: Shoulder Pain and Disability Index
