# Supplementary material for: Emotional distress was associated with persistent shoulder pain after physiotherapy: a prospective cohort study
Source: BMC Musculoskelet Disord. 2018 Aug 22;19:304. doi: 10.1186/s12891-018-2142-3 (PMC6106870; doi:10.1186/s12891-018-2142-3)
Supplement: Supplementary file 1 — Table S1. Characteristics of the group lost to follow-up compared to the study sample. (DOCX 15 kb) [file 12891_2018_2142_MOESM1_ESM.docx]

**Table S1: CHARACTERISTICS OF THE STUDY SAMPLE COMPARED TO THE GROUP LOST TO FOLLOW-UP**

**Study sample n=145, Group lost to follow-up n=64**

| **Characteristics at study onset** | **Study sample**  Mean (SD) | **Group lost to follow-up** Mean (SD) | **Independent t-test** P-value |
| --- | --- | --- | --- |
| Pain intensity (NPRS) | 4.9 (2.3) (missing 18) | 4.7 (2.2) (missing 5) | P = 0.48 |
| Disability (PSFS) | 4.5 (2.0) | 4.8 (2.0) | P = 0.39 |
| Emotional distress (HSCL-25) | 1.6 (0.5) | 1.6 (0.5) | P = 0.93 |
| Age | 44.0 (15.4) | 42.2 (17.7) | P = 0.46 |

|  | **Study sample** | **Group lost to follow-up** | **Chi-square test** P-value |
| --- | --- | --- | --- |
| Sex female / total | 104 / 143 (missing 2) | 46 / 64 | P = 1.0 |

**Patients who provided pain intensity outcome data n=140, patients without outcome data n=69^*^**

| **Characteristics at study onset** | **Patients with pain intensity outcome data**  Mean (SD) | **Patients without outcome data** Mean (SD) | **Independent t-test**  P-value |
| --- | --- | --- | --- |
| Pain intensity (NPRS) | 4.9 (2.3) (missing 16) | 4.8 (2.2) (missing 7) | P = 0.80 |
| Disability (PSFS) | 4.6 (2.0) | 4.6 (2.0) | P = 0.74 |
| Emotional distress (HSCL-25) | 1.6 (0.5) | 1.5 (0.5) | P = 0.66 |
| Age | 43.9 (15.4) | 42.4 (17.5) | P = 0.52 |

|  | **Patients with pain intensity outcome data** | **Patients without outcome data** | **Chi-square test** P-value |
| --- | --- | --- | --- |
| Sex female / total | 99 / 138 (missing 2) | 51 / 69 | P = 0.87 |

^*^Patients lost to follow-up or patients with missing data in the variable of pain intensity after treatment

**Patients who provided disability outcome data n=133, patients without outcome data n=76^*^**

| **Characteristics at study onset** | **Patients with disability outcome data**  Mean (SD) | **Patients without outcome data** Mean (SD) | **Independent t-test**  P-value |
| --- | --- | --- | --- |
| Pain intensity (NPRS) | 4.9 (2.4) (missing 17) | 4.7 (2.1) (missing 6) | P = 0.45 |
| Disability (PSFS) | 4.5 (2.0) | 4.7 (2.0) | P = 0.49 |
| Emotional distress (HSCL-25) | 1.6 (0.5) | 1.6 (0.5) | P = 0.89 |
| Age | 43.6 (15.3) | 43.1 (17.5) | P = 0.84 |

|  | **Patients with disability outcome data** | **Patients without outcome data** | **Chi-square test** P-value |
| --- | --- | --- | --- |
| Sex female / total | 93 / 131 (missing 2) | 57 / 76 | P = 0.65 |

^*^Patients lost to follow-up or patients with missing data in the variable of disability after treatment
